# Pathogenesis and treatment of obesity-related polycystic ovary syndrome

**DOI:** 10.1186/s13048-025-01817-w

**Published:** 2025-11-14

**Authors:** Xiaoling Ouyang, Qi Zhou, Hong Tang, Linxia Li

**Affiliations:** 1 Departments of Gynaecology and Obstetrics, The Seventh People’s Hospital of Shanghai University of Traditional Chinese Medicine, Shanghai, 200137 China; 2 Central Laboratory, The Seventh People’s Hospital of Shanghai University of Traditional Chinese Medicine, Shanghai, 200137 China; 3 Department of Obstetrics and Gynecology, Shanghai General Hospital, Shangahi Jiao Tong University School of Medicine, Shanghai, 200080 China

**Keywords:** Polycystic ovary syndrome, Obesity, Autophagy, Endoplasmic reticulum stress, Chronic low-grade inflammation, Gut microbiota, Hypothalamic-ovarian (HPO) axis, Traditional chinese medicine, Pathogenesis

## Abstract

Polycystic ovary syndrome (PCOS) is a gynecological endocrine disorder affecting 5%–18% of women of reproductive age worldwide. It is characterized by hyperandrogenemia (HA), anovulation, and polycystic ovarian morphology (PCOM), severely impacting women’s reproductive and metabolic health. Obesity has become increasingly common among PCOS patients in recent years. Obesity can further exacerbate the metabolic and reproductive dysfunctions of PCOS through mechanisms such as insulin resistance (IR) and chronic low-grade inflammation. It may even have adverse effects on mental health. However, the specific pathogenesis and effective therapeutic targets of PCOS with obesity remain incompletely understood. This review presents a narrative review of recent research, focusing on the molecular mechanisms that drive autophagy in the context of obesity associated with polycystic ovary syndrome (PCOS), endoplasmic reticulum stress (ERS), gut microbiota imbalances, and disruptions in the hypothalamic-pituitary-ovarian (HPO) axis. It also explores corresponding therapeutic strategies. The aim is to provide fresh perspectives and insights for future mechanistic research and clinical interventions in this field.

## Introduction

Polycystic ovary syndrome (PCOS) is one of the most common endocrine disorders among women of reproductive age, affecting approximately 5%–18% of this population worldwide [1, 2]. It is primarily defined by the presence of hyperandrogenemia (HA), ovulatory dysfunction, and polycystic ovarian morphology (PCOM). The clinical manifestations of PCOS are notably diverse and are often accompanied by comorbidities such as glucose and lipid metabolism disorders, obesity, cardiovascular diseases, and other systemic conditions [3].

Obesity is notably prevalent among PCOS women, with an estimated 49%-80% of affected individuals being classified as overweight or obese [4–6]. It is both a common comorbidity and a significant pathogenic factor in PCOS [7, 8]. Clinical studies demonstrate that obese PCOS women (BMI ≥ 25 kg/m²) typically experience more severe endocrine disturbances, including HA, as well as greater abnormalities in glucose and lipid metabolism, such as insulin resistance (IR), increased visceral fat, and reproductive dysfunction, compared to non-obese PCOS patients [9, 10]. The high prevalence of obesity in PCOS is unlikely to be coincidental; increasing evidence suggests that it contributes actively to disease progression through intertwined metabolic and endocrine disruptions.

Mechanistically, visceral adipose tissue (VAT) is a key hub linking obesity to PCOS pathophysiology. Excessive release of free fatty acids (FFAs) from VAT induces systemic IR [11, 12]. The subsequent hyperinsulinemia suppresses sex hormone-binding globulin (SHBG) synthesis, increasing circulating levels of free testosterone [13]. Furthermore, insulin and insulin-like growth factor 1 (IGF-1) have been demonstrated to promote testosterone synthesis by activating insulin receptors and insulin-like growth factor 1 receptors (IGF-1R) on ovarian theca cells (TCs), which subsequently enhances the enzymatic activity of cytochrome P450c17α (CYP17A1) [14]. Elevated androgen levels further aggravate metabolic homeostasis by promoting the differentiation of preadipocytes into visceral adipocytes via androgen receptor (AR) signaling, while simultaneously inhibiting lipolysis in subcutaneous fat [10, 15]. This reciprocal interaction between HA and IR may synergistically promote visceral fat accumulation, forming a self-reinforcing cycle [16].

Obesity also disrupts neuroendocrine signaling, particularly the hypothalamic-pituitary-ovarian (HPO) axis. In adipose tissue, aromatase (CYP19A1) converts excess androgens into estrogens, which exert negative feedback on gonadotropin-releasing hormone (GnRH) secretion. This results in an altered luteinizing hormone (LH) to follicle-stimulating hormone (FSH) ratio, contributing to ovulatory dysfunction and menstrual irregularities.

Additionally, obesity contributes to ovarian dysfunction through multiple molecular mechanisms. Within the ovary, the interplay between HA and IR initiates a series of pathological events, including endoplasmic reticulum stress (ERS) and autophagy dysfunction in ovarian granulosa cells (GCs). These cellular alterations culminate in GC dysfunction and apoptosis, ultimately impairing folliculogenesis [17–19]. The excessive accumulation of adipose tissue contributes to a systemic inflammatory response and disrupts the composition of the gut microbiota. These alterations, mediated by the secretion of various adipokines, synergistically exacerbate metabolic and reproductive dysfunctions in PCOS [16, 20].

Collectively, obesity is increasingly recognized as a major contributor to PCOS progression. However, the downstream pathways—such as aberrant autophagy, ER stress, chronic inflammation, gut dysbiosis, and HPO axis disruption—remain to be fully elucidated. Although modern medicine and traditional Chinese medicine (TCM) show therapeutic complementarity, elucidating their shared molecular targets is a prerequisite for developing precision interventions.

This narrative review is based on literature retrieved from PubMed, Web of Science, and CNKI between 2018 and 2025, using keywords including “PCOS,” “obesity,” “autophagy,” “endoplasmic reticulum stress,” “inflammation,” “gut microbiota,” “HPO axis,” and “traditional Chinese medicine”. Relevant preclinical and clinical studies were included to provide mechanistic and therapeutic insights into obesity-related PCOS. In this review, we summarize the underlying mechanisms and therapeutic strategies, aiming to offer new insights into the individualized treatment of obesity-associated PCOS.

## Pathogenesis mechanism

### Autophagy

Autophagy is a cellular process essential for maintaining homeostasis by degrading damaged organelles and proteins via lysosomes. It encompasses three primary types: macroautophagy, microautophagy, and chaperone-mediated autophagy (CMA). Macroautophagy is particularly important in the ovary, where it plays a pivotal role in regulating oocyte development, follicular growth, and atresia. In PCOS, especially in the context of obesity, dysregulated autophagy disrupts GCs and TCs function, contributing to follicular arrest and anovulation [21]. Notably, this dysregulation is cell-type specific: excessive autophagy is often observed in GCs, while autophagic impairment is more common in TCs [22–27].

IR is tightly linked to altered autophagy in PCOS, particularly in GCs. In PCOS patients, reduced ATG7 levels correlate negatively with HOMA-IR, indicating disrupted autophagy [28]. IR promotes mitochondrial dysfunction and oxidative stress, activating mitophagy via the mitochondrial unfolded protein response (UPR^mt). This leads to GC dysfunction and premature ovarian aging [29]. Conversely, excessive autophagy activation—especially via high mobility group box 1 (HMGB1)—negatively regulates insulin signaling by suppressing IRS-1 and inhibiting the PI3K/AKT pathway, creating a self-perpetuating loop between IR and imbalance between autophagic activation and degradation [30, 31]. This bidirectional interaction is a critical driver of metabolic and reproductive dysfunction in obese PCOS.

HA also induces excessive autophagy in GCs via multiple converging molecular mechanisms. It suppresses the PI3K/AKT/mTOR axis and promotes ROS accumulation—two key pathways known to trigger autophagic activation [32–34]. These changes are consistently observed in clinical and animal models, evidenced by elevated LC3-II/LC3-I ratios and reduced p62 expression [23–25]. Additionally. BORC complex subunit BOP1 (BOP1) overexpression triggers a nucleolar stress response, further inhibiting mTOR signaling via p53, thereby exacerbating autophagic activity [23]. In parallel, CISD2 upregulation suppresses mitophagy, leading to ROS buildup and mitochondrial dysfunction, which disrupts GC survival [35]. Wnt5a has emerged as a potential modulator capable of restoring autophagic homeostasis in HA-induced PCOS. By downregulating PI3K/AKT/mTOR signaling, Wnt5a attenuates excessive autophagy and alleviates GC dysfunction in experimental models [36]. Although most evidence centers on GCs, HA disrupts autophagy in peripheral metabolic tissues. In skeletal muscle cells, HA impairs glucose uptake by inhibiting the mTORC1-autophagy axis, resulting in impaired glucose uptake and aggravated IR [37]. Additionally, the mitophagy-related gene MAP1LC3A is positively correlated with serum testosterone in PCOS patients and may serve as a biomarker of ovulatory dysfunction [38]. These findings underscore the role of HA in disrupting both canonical autophagy and mitophagy across multiple tissues, thereby promoting both ovarian and metabolic dysfunction in obese PCOS.

FFAs in obesity-related PCOS disrupt autophagy homeostasis in both TCs and GCs, worsening follicular and metabolic dysfunction. In TCs, FFAs impair autophagosome–lysosome fusion, leading to p62 accumulation. This suppression is associated with upregulated CYP17A1 and PAI-1 expression, which enhance androgen production via ROS/p38 and JNK signaling [22]. In GCs, FFAs stimulate the secretion of adipokine chemerin, which excessively activates autophagy in GCs by inhibiting the PI3K/Akt/mTOR pathway [39–41]. The resulting excessive autophagy activation leads to mitochondrial damage and cellular stress, thereby impairing folliculogenesis. This pathophysiological axis links lipid metabolism, autophagy dysregulation, and ovarian dysfunction. Together, FFAs induce divergent autophagic phenotypes—suppressed flux in TCs and excessive activation in GCs—via distinct molecular pathways. These effects collectively disrupt follicular integrity and contribute to reproductive and metabolic abnormalities of obese PCOS.

In patients with PCOS and obesity, autophagy is primarily marked by excessive activation of ovarian GCs and impaired autophagy in TCs. This dysregulation is driven by several factors, including IR, HA, and abnormal lipid metabolism. These factors contribute to abnormalities in follicular development and a cycle of metabolic dysfunction. Future research should aim to dissect cell-specific autophagic pathways and develop targeted interventions to restore autophagic balance and improve reproductive-metabolic outcomes in PCOS.

### Chronic low-grade inflammation

Chronic low-grade inflammation (CLGI), characterized by the persistent elevation of pro-inflammatory mediators such as CRP, IL-6, and TNF-α, is a defining feature of obesity-associated PCOS [42–47]. Compared with non-obese patients with PCOS, those with obesity demonstrated more severe inflammatory responses, including increased levels of IL-6, TNF-α, neutrophil-to-lymphocyte ratio (NLR), high-sensitivity C-reactive protein (hs-CRP), and mean platelet volume (MPV) levels [48, 49]. These systemic alterations reflect a sustained inflammatory milieu driven by both metabolic stress and endocrine perturbations.

At the molecular level, CLGI in PCOS is orchestrated by aberrant immune cell activation, inflammasome signaling, and tissue-specific inflammatory responses. In the liver, overnutrition suppresses AMP-activated protein kinase (AMPK) activation, leading to activation of the NLRP3 inflammasome and IL-1β maturation—key events in initiating hepatic inflammation. Simultaneously, IRS1 Ser307 phosphorylation further links metabolic stress to immune dysregulation [50]. In the endometrium, the reduced abundance of CD56^+^ NK cells and CD163^+^ M2 macrophages compromises local immune tolerance, favoring a pro-inflammatory microenvironment [51, 52]. In ovarian GCs, inflammatory stimuli such as advanced glycation end-products (AGEs) and high glucose activate the p38 MAPK pathway, enhancing secretion of IL-6 and TNF-α and impairing follicular development [53].

In addition to canonical immune pathways, endocrine-immune crosstalk reinforces the inflammatory state. While IR and HA are known to contribute to inflammation, emerging evidence suggests that inflammation may independently drive metabolic and reproductive dysfunction in PCOS. For example, elevated IL-15 levels in patients and animal models activate p38 and JNK signaling in GCs, stimulating pro-inflammatory cytokine production and exacerbating steroidogenic imbalance [54]. Furthermore, adipokines such as chemerin accumulate in the follicular fluid and directly impair oocyte competence by disrupting intracellular signaling [40, 41, 55].

Dysregulated lipid metabolism further exacerbates CLGI by altering adipose tissue homeostasis and immune balance. PCOS patients frequently exhibit dyslipidemia, typified by elevated triglyceride (TG) and low-density lipoprotein cholesterol (LDL-C) levels alongside reduced high-density lipoprotein cholesterol (HDL-C). These lipid abnormalities positively correlate with inflammatory markers such as CRP and the systemic immune-inflammation index (SII) [56, 57]. Increased leukocyte counts and immune cell activation further reflect this link [58]. Notably, compensatory mechanisms may exist. In PCOS mouse models, the peptide hormone adropin promotes M2 macrophage polarization and eNOS/PPARγ-mediated browning of white adipose tissue, thereby mitigating inflammation and restoring metabolic homeostasis [59].

Obesity may exacerbate CLGI in PCOS through the promotion of HA, IR, and lipid metabolism disorders, which in turn aggravate metabolic dysfunction. However, inflammation may also function as an independent pathogenic factor in PCOS, potentially contributing to disease heterogeneity between obese and non-obese patients. Future research should focus on clarifying causal relationships and developing therapeutic strategies targeting the metabolism-inflammation axis.

### Endoplasmic reticulum stress

Endoplasmic reticulum stress (ERS) is a cellular adaptive response triggered by disruptions in ER homeostasis, affecting protein folding, calcium balance, and lipid processing. Moderate levels of ERS are essential for oocyte maturation [60]. However, excessive or sustained ERS has been demonstrated to significantly exacerbate the pathological progression of PCOS [61].

Clinical and experimental studies have consistently demonstrated heightened ERS in PCOS, particularly in ovarian GCs. Elevated levels of ERS markers, such as glucose-regulated protein 78 (GRP78) and activating transcription factor 4 (ATF4), have been identified in the GCs of PCOS patients [62, 63]. Animal models further corroborate these findings, showing the activation of key ERS pathways, including inositol-requiring enzyme 1α (IRE1α) and protein kinase RNA-like endoplasmic reticulum kinase (PERK), correlating with increased GC apoptosis and impaired follicular development [61, 64].

A primary upstream trigger of ERS in PCOS is metabolic overload, particularly FFA accumulation associated with obesity. Elevated FFAs activate PERK and IRE1α signaling in GCs, inducing apoptosis and steroidogenic dysfunction. Simultaneously, FFAs upregulate CYP17A1 and PAI-1 via the ROS/p38 and JNK axes, enhancing androgen synthesis and amplifying ERS. Inflammatory cytokines released from adipose tissue, such as IL-6 and TNF-α, further intensify ER stress signaling, indicating cross-talk between metabolic and immune perturbations [61–63, 65, 66].

Hyperglycemia and glucose toxicity, frequently observed in insulin-resistant PCOS patients, represent an additional ERS trigger. Persistent high glucose conditions exacerbate oxidative damage and upregulate ERS markers such as ATF4 and XBP1 in GCs, promoting apoptosis and compromising follicular integrity [65, 67]. This hyperglycemia-induced ERS further disrupts systemic insulin sensitivity, creating a detrimental feedforward loop that aggravates metabolic dysfunction.

HA exacerbates ERS through oxidative and inflammatory mechanisms. Androgen excess activates the NOX4/ROS axis, leading to sustained PERK–ATF4 signaling and induction of the LINC00173–HRK apoptotic pathway, while concurrently inhibiting PI3K/Akt-mediated survival signaling [64, 68]. In parallel, HA enhances the IRE1α-TXNIP-NLRP3 inflammasome activation, leading to pyroptosis and local inflammation in GCs [69, 70]. Additionally, HA promotes lipid peroxidation and ferroptosis in GCs, while systemically inducing ERS in uterine smooth muscle, impairing contractility and implantation, and in pancreatic β-cells, driving hyperinsulinemia [71–74].

In conclusion, ERS in PCOS represents a critical downstream effector of integrated metabolic and endocrine insults. By disrupting GC survival, steroidogenesis, and tissue homeostasis, sustained ERS contributes to follicular arrest, chronic inflammation, and systemic dysfunction. Therapeutic strategies targeting ERS-related pathways may offer novel avenues for restoring reproductive and metabolic balance in PCOS.

### Gut microbiota dysbiosis

Gut microbiota plays a central role in host metabolic homeostasis, immune regulation, and endocrine signaling. Through fermentation of dietary fibers, it produces short-chain fatty acids (SCFAs), maintains intestinal barrier integrity, and regulates tryptophan and bile acid metabolism. These microbial metabolites serve as signaling molecules that regulate systemic inflammation, glucose metabolism, and hormonal balance.

In obese PCOS patients, gut dysbiosis is a common feature, marked by reduced α-diversity, depletion of beneficial microbes (e.g., Bacteroidetes, Firmicutes), and increased abundance of pro-inflammatory taxa (Proteobacteria, Fusobacterium, Prevotella) [75, 76]. The decline in butyrate-producing bacteria and reduced SCFA levels, weakening the intestinal barrier and facilitating lipopolysaccharide (LPS) leakage into the circulation. LPS activates the TLR4/NF-κB pathway, inducing CLGI. Moreover, SCFAs deficiency promotes epitranscriptomic changes, such as m6A methylation of FOSL2, which activates the NLRP3 inflammasome and enhances IL-1β secretion [49].

In parallel, the altered microbial composition impairs lipid and bile acid metabolism. Enrichment of Megamonas and Dialister further contributes to IR via disruptions in fatty acid and sphingolipid signaling pathways [77]. Additionally, a decrease in secondary bile acids, such as GDCA and TUDCA, impairs IL-22 secretion, exacerbating local ovarian inflammation and metabolic imbalance [78].

Importantly, HA aggravates gut dysbiosis through AR-mediated regulation of the FKBP5 gene, promoting DNA hypomethylation and microbial imbalance. This HA-microbiota feedback loop further impairs metabolic homeostasis. Androgens also reshape bile acid profiles and intestinal immunity, further shifting microbial ecology toward pro-inflammatory taxa [79]. This creates a vicious cycle wherein gut dysbiosis and HA reinforce each other, jointly driving endocrine and metabolic dysfunction.

In summary, gut microbiota dysbiosis in obese PCOS is both a cause and consequence of metabolic imbalance, mediated through disrupted SCFA and bile acid metabolism, loss of barrier integrity, and androgen-induced microbial shifts. These findings highlight the potential of microbiota-centered therapies—such as probiotics, prebiotics, and fecal microbiota transplantation—to restore host–microbiota homeostasis and break the pathophysiological cycle of obesity-related PCOS.

### Hypothalamic-pituitary-ovarian (HPO) axis

The HPO axis plays a central role in regulating reproductive function by orchestrating the secretion of LH and FSH through the pulsatile release of GnRH. This process coordinates follicular development and sex hormone synthesis. Under physiological conditions, estradiol exerts positive feedback to induce a preovulatory LH surge, while negative feedback maintains hormonal homeostasis [80].

In PCOS, the frequency of GnRH pulses is pathologically elevated, preferentially enhancing LH secretion over FSH. This disrupts the FSH-mediated induction of CYP19A1 (aromatase), impairing the conversion of androgens to estrogens, and resulting in HA and follicular arrest—core features of PCOS pathophysiology [81].

Obesity exacerbates neuroendocrine dysregulation through multiple interrelated mechanisms. Leptin resistance and chronic hyperleptinemia overstimulate GnRH neurons via kisspeptin signaling, promoting excessive LH release and subsequent androgen overproduction [80, 82, 83]. Concurrently, hyperinsulinemia activates AgRP neurons and increases the expression of neuropeptide Y (NPY) and GABA, which synergistically upregulate hypothalamic kisspeptin and GnRH expression, further aggravating LH hypersecretion [84–86]. Altered gut microbiota—characterized by reduced levels of secondary bile acids such as glycodeoxycholic acid (GDCA) and tauroursodeoxycholic acid (TUDCA)—impair the bile acid-IL-22 signaling axis, diminishing hypothalamic sensitivity to androgen feedback and accelerating GnRH pulse generation [78].

These convergent pathways disrupt the LH/FSH ratio, amplify ovarian androgen output, and impair folliculogenesis. Ultimately, obesity-induced neuroendocrine reprogramming intensifies HPO axis dysfunction, contributing to anovulation and reproductive failure in PCOS.

**Fig. 1:**
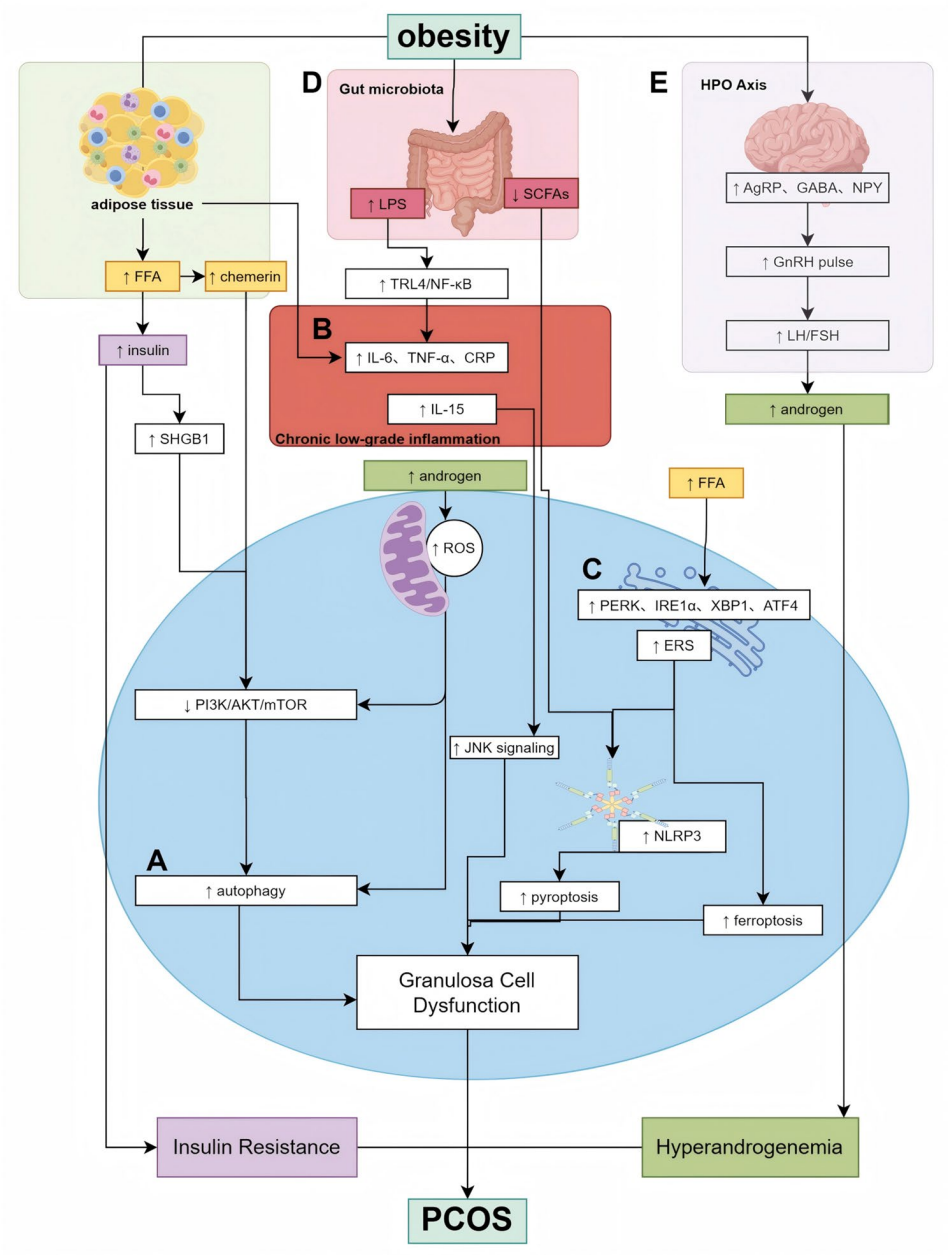
Mechanisms underlying obesity-associated PCOS. (**A**) Autophagy dysregulation: Obesity-induced IR and HA promote oxidative stress, impairing autophagic flux in GCs and disrupting follicular development. (**B**) Chronic low-grade inflammation: Adipocyte-derived pro-inflammatory cytokines (e.g., TNF-α, IL-6) activate immune pathways such as NF-κB and NLRP3 inflammasome, contributing to ovarian inflammation and androgen excess. (**C**) ER stress: Obesity and metabolic imbalance lead to ER stress, triggering unfolded protein response (UPR) and exacerbating cellular apoptosis, hormone imbalance, and inflammation. (**D**) Gut microbiota dysbiosis: High-fat diet-induced obesity alters gut microbial composition and reduces SCFA production, increasing intestinal permeability and LPS translocation, which enhances systemic inflammation and insulin resistance. (**E**) HPO axis disruption: Obesity impairs GnRH pulsatility and disrupts LH/FSH secretion, resulting in abnormal folliculogenesis and anovulation, further aggravating PCOS symptoms The Fig. 1 is by Figdraw (https://www.figdraw.com)

In summary, obesity-driven alterations in metabolic, endocrine, and microbial pathways converge to intensify HPO axis dysfunction in PCOS Figure 1. Further elucidation of the molecular crosstalk between adipokines, central neuronal circuits, and peripheral metabolites may reveal novel targets for neuromodulatory interventions aimed at restoring reproductive endocrine homeostasis in obese PCOS patients.

## Treatment

The treatment of obesity-associated PCOS requires a comprehensive, multi-targeted strategy due to the complexity of its pathophysiology. Current therapeutic approaches aim to restore metabolic and reproductive homeostasis by modulating autophagy, alleviating ERS, reducing chronic inflammation, remodeling gut microbiota, and regulating the HPO axis. Integrative therapies that combine TCM and Western medicine have shown synergistic benefits in improving metabolic profiles and ovarian function Table 1. However, the underlying mechanisms remain incompletely understood, and further research is essential to support their clinical translation.

### Autophagy modulation

Dysregulated autophagy contributes to follicular arrest and metabolic dysfunction in obesity-associated PCOS. Preclinical studies suggest that pharmacological agents, TCM, and acupuncture modulate key autophagy-related pathways, potentially restoring cellular homeostasis.

#### Pharmacological therapy

Melatonin promotes mitophagy via upregulation of Clock genes, while the combination of clomiphene and dexamethasone attenuates GC apoptosis by targeting the ROS–JNK/MAPK–p21 axis and restoring autophagic flux [87, 88].

#### Chinese herbal formulas and monomers

Chinese herbal interventions modulate autophagy via multiple signaling pathways in both GCs and endometrial cells. Cangfu Daotan Decoction and Guizhi Fuling Wan (GFW) inhibit autophagy via the FOXK1/Wnt/β-catenin and PI3K/AKT/mTOR axis, respectively [30, 89–91]. Bushen Huoluo Decoction and Yishen Jianpi Yangxue Tongli Formula restore autophagy balance by targeting exosomal miR-30a-5p/SOCS3/mTOR and PI3K/AKT1/FOXO1 pathways [92, 93]. Chaonangqing Formula targets GATA3 and MYCT1 to regulate both apoptosis and autophagy in GCs [94]. Monomers such as berberine, curcumin, and protocatechuic acid restore autophagy and alleviate insulin resistance via AMPK/mTOR and NF-κB pathways [95–97]. Naringenin and morin enhance both apoptosis and autophagy markers in endometrial cells, alleviating hyperplasia [98]. These compounds offer multi-level regulatory effects on autophagy and represent potential adjunctive therapies for PCOS in obese individuals.

#### Acupuncture

Electroacupuncture (EA) dynamically adjusts autophagy by inhibiting or enhancing PI3K/AKT/mTOR signaling, improving hyperandrogenism and insulin sensitivity in PCOS models [99–102].

In summary, targeting autophagy represents a mechanistic avenue for PCOS intervention. While diverse modalities converge on PI3K/AKT/mTOR and oxidative stress-related pathways, current evidence is predominantly preclinical, and further clinical validation is essential.

### Regulation of chronic low-grade inflammation

CLGI, driven by adipokines, IR, and oxidative stress, plays a central role in the pathogenesis of obesity-related PCOS. Targeting inflammatory pathways has demonstrated potential in alleviating both metabolic abnormalities and reproductive dysfunction.

#### Lifestyle interventions

Lifestyle interventions remain the first-line strategy. Caloric restriction and dietary changes lower proinflammatory cytokines, such as IL-6 and TNF-α, improving insulin sensitivity and ovulatory outcomes [103]. In severely obese patients (BMI ≥ 35 kg/m²), bariatric surgery further reduces adipose inflammation and enhances reproductive function [104].

#### Pharmacological therapy

Several pharmacologic agents modulate inflammation by regulating lipid metabolism and suppressing cytokine production. EE/DRSP reduces ferritin levels, indirectly alleviating IR-related inflammation [105]. Crocin and atorvastatin improve lipid profiles and inhibit NF-κB-mediated cytokine expression, restoring endocrine function [106, 107].

#### Chinese herbal formulas and monomers

TCM regulates inflammation and exerts anti-inflammatory effects through multiple pathways. Heqi San, Bailing Capsule, and QGW suppress proinflammatory cytokines via inhibition of NF-κB or activation of the IRS1/PI3K/AKT and Nrf2-HO-1 signaling [108–110]. Zishen Qingre Lishi Huayu recipe and the combination of GFW and rosiglitazone also reduce CRP, IL-6, and IL-1β expression in ovarian tissues, although their precise mechanisms remain unclear [111, 112].

Among monomers, curcumin and ostiole inhibit NF-κB signaling through the TLR4/MyD88 pathway or the Nrf2–Foxo1–GSH axis, respectively [113, 114]. Resveratrol attenuates NLRP3-mediated pyroptosis to support follicular development [115]. Although Althaea officinalis extract and astaxanthin combined with curcumin show additive anti-inflammatory effects, the precise mechanisms remain unclear [116, 117].

In summary, these herbal formulas and monomers converge mainly on the NF-κB and NLRP3 pathways, offering mechanistic insight into their anti-inflammatory potential in PCOS. Nonetheless, their efficacy requires confirmation in well-designed clinical studies to enable clinical translation.

### Alleviation of endoplasmic reticulum stress

ERS contributes to the metabolic and reproductive dysfunctions in PCOS by promoting GC apoptosis and impairing follicular development. Therapeutic modulation of ERS has emerged as a promising strategy for restoring ovarian homeostasis.

#### Pharmacological interventions

Pharmacological agents alleviate ERS in PCOS by targeting unfolded protein response (UPR) pathways. Metformin downregulates ER stress markers such as CHOP, thereby reducing GC apoptosis and promoting follicular development [19]. Irisin inhibits IRE1α signaling to improve ovarian dysfunction [69], while adrenomedullin (ADM) exerts anti-apoptotic and anti-inflammatory effects via PI3K/Akt and PPAR-γ activation [118]. These agents converge on UPR suppression and mitochondrial protection, highlighting their potential to restore GC homeostasis.

#### Chinese herbal formulas and monomers

TCM interventions alleviate ERS through stress-related pathways. Bushen Jieyu Tiaochong Decoction and Kunling Pill mitigate ERS-induced apoptosis by suppressing PERK/ATF4/CHOP and IRE1α pathways in GCs [119, 120]. At the monomer level, curcumin blocks the IRE1α-XBP1 axis while activating PI3K/AKT [119, 121]. Astaxanthin reduces GRP78 and CHOP expression, though its metabolic effects require further validation [122].

#### Acupuncture

EA alleviates ER stress in GCs primarily through inhibition of the PERK/eIF2α/ATF4/CHOP pathway and may also exert enhanced autophagy, contributing to organelle quality control [101, 102].

ERS-targeted therapies in PCOS primarily act through PERK and IRE1α signaling to reduce GC apoptosis and restore ovarian function. Although pharmacological agents, TCM, and acupuncture exhibit convergent effects via UPR inhibition and mitochondrial protection, most findings remain preclinical, underscoring the need for further clinical validation.

### Gut microbiota remodeling

Gut microbiota dysbiosis contributes to the metabolic, inflammatory, and reproductive abnormalities observed in obesity-related PCOS. Modulating the gut microbiota has thus emerged as a promising therapeutic strategy. This section outlines current strategies—nutritional, pharmacological, and TCM-based—that aim to restore microbial balance and alleviate PCOS.

#### Nutritional intervention

Dietary modification improves PCOS symptoms by modulating the gut microbiota and reducing systemic inflammation. Clinical studies have shown reductions in BMI, fasting blood glucose (FBG), total cholesterol (TC), and TG, alongside increased abundance of beneficial bacteria (e.g., Bacteroidetes) and decreased Firmicutes [123]. These findings support nutritional intervention as a first-line strategy to restore microbial and metabolic homeostasis.

#### Pharmacological therapy

Metformin enhances SCFA production, improves insulin sensitivity, and modifies gut microbiota composition. Combined with probiotics or calorie restriction, it shows synergistic effects on glycemic control and hormone regulation [124, 125]. These benefits are mediated through the interplay between microbial metabolites and host metabolism.

0.3.4.3 Chinese Herbal Formulas and Monomers.

TCM formulations improve gut microbial diversity and metabolic parameters. Buzhong Yiqi Decoction and Heqi San promote microbial balance and ameliorate metabolic dysfunction [108, 126]. Jiawei Qigong Pill and Bailing Capsule attenuate inflammation and IR by inhibiting the LPS–TLR4 signaling pathway [109, 127]. At the monomer level, Antrodia camphorata polysaccharides enhance intestinal barrier integrity and regulate the relative abundance of *Bacteroides*, *Firmicutes*, and *Verrucomicrobia*, contributing to systemic improvement [128].

#### Acupuncture

EA alters gut microbiota composition, particularly the abundance of *Tenericutes* and *Prevotella_9*, and improves brown adipose tissue function [129]. Collectively, these changes enhance metabolic and reproductive outcomes in PCOS models.

Interventions targeting gut microbiota—via diet, drugs, TCM, or acupuncture—modulate microbial composition, reinforce intestinal barrier integrity, and suppress inflammation. These findings underscore the close interplay between gut microbiota, metabolic regulation, and reproductive function in obesity-associated PCOS, highlighting the gut as a promising therapeutic target.

### Regulation of the HPO axis

HPO axis dysregulation is a central driver of hormonal imbalance, anovulation, and metabolic disturbances in PCOS. Therapeutic modulation of this axis shows promise, particularly in obese phenotypes. This section reviews pharmacological, TCM-derived, and acupuncture-based strategies aimed at restoring HPO axis homeostasis.

#### Pharmacological therapy

The combination of metformin and Glucagon-Like Peptide-1 (GLP-1) receptor agonist exenatide improves insulin sensitivity and suppresses ovarian androgen production. These agents may help restore HPO axis function by rebalancing gonadotropin secretion, particularly the LH/FSH ratio, and improving systemic metabolic profiles in obese PCOS patients [130].

#### Traditional Chinese medicine monomers

Flavonoids from *Eucommia ulmoides* and crocetin exert central and peripheral regulation on the HPO axis. Total flavonoids of *Eucommia ulmoides* leaves (TFEL) improve hormone levels and ovarian/pancreatic histopathology, while crocetin modulates hypothalamic kisspeptin neurons—enhancing AVPV-kisspeptin and inhibiting ARC-kisspeptin—to improve ovulatory function [131, 132]. These findings highlight their potential in endocrine reprogramming.

#### Acupuncture

Cheek acupuncture promotes ovulation and endometrial receptivity by reflexively modulating HPO axis activity [133]. Although the precise mechanisms remain unclear, it is hypothesized to influence hypothalamic neuroendocrine signaling via somatic–visceral reflex pathways.

In summary, therapies targeting the HPO axis—including metabolic agents, TCM monomers, and acupuncture—aim to restore axis homeostasis and improve reproductive and metabolic outcomes in obesity-related PCOS.

**Table 1:** Summary of therapeutic interventions for obesity-related PCOS

	Category	Intervention	Mechanism	Evidence Level
Autophagy modulation	Pharmacological	Melatonin	↑Mitophagy via Clock gene regulation	Preclinical
		Clomiphene + Dexamethasone	↓Apoptosis/autophagy via ROS-JNK/MAPK-P21	Preclinical
	TCM Formulas	Cangfu Daotan Decoction	↓Autophagy via FOXK1/Wnt/β-catenin	Preclinical
		Guizhi Fuling Wan	↓Autophagy via H19/miR-29b-3p/mTOR	Preclinical
		Bushen Huoluo Decoction	Regulates miR-30a-5p/SOCS3/mTOR/NLRP3	Preclinical
		Chao Nang Qing	↑Autophagy/apoptosis via GATA3	Preclinical
	TCM Monomers	Berberine + Metformin	AMPK/AKT/mTOR pathway regulatio	Preclinical
		Protocatechuic Acid	PI3K-mediated ↓ROS/autophagy	Preclinical
		Nanocurcumin	Modulates miR-223-3p/NF-κB autophagy	Preclinical
		Naringenin/Morin	↑Autophagic apoptosis	Preclinical
	Acupuncture	Electroacupuncture	↓PI3K/AKT/mTOR pathway	Preclinical
Regulation of Chronic Low-Grade Inflammation	Lifestyle Modification	Diet-induced weight loss	↓Inflammatory markers (CRP, IL-6, TNF-α)	Clinical
	Pharmacological	EE/DRSP	↑Ferritin levels (potentially related to inflammation)	Clinical
		Atorvastatin	↓Adipose tissue dysfunction and inflammation markers (ASP, IL-6, MCP-1)	Clinical
		Crocin	↓Inflammatory markers (IL-6, TNF-α)	Clinical (RCT)
	TCM Formulas	HeQi San	↓Inflammatory markers (IL-6, TNF-α)	Preclinical
		Bailing Capsule	↓intestinal-derived LPS-TLR4 inflammatory pathway	Preclinical
		Qi Gong Wan	↑Nrf2/HO-1/Cyp1b1 pathway	Preclinical
		Zishen Qingre Lishi Huayu Recipe	↓IL-6, IL-1β, and CRP	Preclinical
		Guizhi Fuling Wan combined with rosiglitazone	↓PI3K/AKT/NF-κB and ↑Nrf2/HO-1 pathways	Preclinical
	TCM Monomers	Curcumin	↓TLR4/MyD88/NF-κB signaling pathway	Preclinical
		Osthole	↓Nrf2-Foxo1-GSH-NF-κB signaling pathway	Preclinical
		Resveratrol	↓NLRP3/GSDMD/Caspase-1-mediated pyroptosis	Preclinical
Alleviation of Endoplasmic Reticulum Stress	Pharmacological	adrenomedullin (ADM)	↑PI3K/Akt1 and PPAR-γ pathways	Preclinical
	TCM Formulas	Bushen Huatan Granules and Kunling Wan	↓GRP78, C/EBP, p-IRE-I, ATF4	Preclinical
	TCM Monomers	Curcumin	↓IRE1α-XBP1 pathway	Preclinical
		astaxanthin	↓ GRP78, CHOP, XBP1, ATF4, ATF6 and DR5	Clinical (RCT)
Gut Microbiota Remodeling	Lifestyle Modification	Nutritional Intervention	↓ B. vulgatus, F. prausnitzii, E. rectale, B. uniformis, and Roseburia intestinalis; ↑A. hadrus, F. plautii, Lactobacillus ruminis, Bifidobacterium breve, and Oligotropha carboxidovorans	Clinical
		probiotics	↑Abundance of gut microbiota	Clinical
	Pharmacological	Metformin	↑beneficial bacteria	Clinical
	TCM Formulas	Buzhong Yiqi Prescription	↑[Eubacterium]_rectale_group, Escherichia-Shigella, and Fusicatenibacter	Clinical
		Qi Gong Wan	↑the diversity of intestinal flora, ↑the number of intestinal probiotics	Clinical
	TCM Monomers	Antrodia cinnamomea polysaccharide (ACP)	↑the α-diversity and modulated the abundance of phyla (Bacteroidetes, Firmicutes, and Verrucomicrobia) and genera (Lactobacillus, Helicobacter, Akkermansia, Oscillospira, Coprococcus, Roseburia, Blautia, and Allobaculum)	Preclinical
	Acupuncture	Electroacupuncture	Tenericutes at the phylum level and Prevotella_9 at the genus level	Preclinical
Regulation of the HPO Axis	Pharmacological	Metformin Combined with Exenatide	↑HPO axis	Clinical
	TCM Monomers	total flavonoids from Eucommia ulmoides Oliv. leaves	↓Kiss1/IGF-1/LEPR/AR in the HPO axis	Preclinical
		Crocetin	↑AVPV-kisspeptin, ↓ARC-kisspeptin	Preclinical
	Acupuncture	cheek acupuncture	Regulate HPO axis	Clinical

## Discussion

PCOS is a common endocrine–metabolic disorder in reproductive-aged women. Obesity is not only a common comorbidity but also an important contributor to its pathogenesis. It exacerbates reproductive and metabolic dysfunction through interconnected mechanisms, including dysregulated autophagy, ERS, chronic low-grade inflammation, gut microbiota imbalance, and HPO axis disruption.

Among these mechanisms, impaired autophagy in GCs and TCs contributes to follicular arrest by disrupting ovarian microenvironmental stability. ERS, often driven by lipotoxicity, IR, and HA, promotes GC apoptosis and impairs ovulation. CLGI, maintained by adipokine secretion and metabolic stress, further aggravates endocrine and immune dysfunction. Gut microbiota also plays a regulatory role by affecting systemic metabolism, immune balance, and steroid hormone levels. Additionally, HPO axis dysfunction—mediated by leptin resistance, neuropeptide imbalance, and disrupted steroid feedback—links obesity to ovulatory disturbances.

Lifestyle intervention remains the cornerstone of treatment for obese PCOS patients, with strong evidence supporting its benefits on metabolic and reproductive outcomes. Complementary strategies, particularly TCM and acupuncture, show potential in modulating key pathological processes such as autophagy, CLGI, ERS, and HPO axis dysfunction. These therapies offer multi-target regulation and may serve as adjuncts to conventional treatment. Integrated strategies that combine TCM with Western medicine hold potential for individualized management.

However, while TCM-based interventions demonstrate mechanistic promise, their clinical efficacy remains inadequately validated within the framework of evidence-based medicine. Current findings are primarily derived from preclinical studies or small-scale clinical trials, often with methodological limitations. Future research should prioritize large-scale, high-quality randomized controlled trials, adopt standardized outcome measures, and further explore molecular mechanisms to support the integration of TCM into modern PCOS treatment.

Overall, obesity plays a pivotal role in the pathophysiology of PCOS, and interventions targeting its core mechanisms may enable more personalized and evidence-based management.

## Data Availability

No datasets were generated or analysed during the current study.

## Abbreviations

PCOS: Polycystic ovary syndrome
HA: Hyperandrogenemia
PCOM: Polycystic ovarian morphology
BMI: Body mass index
IR: Insulin resistance
VAT: Visceral adipose tissue
FFAs: Free fatty acids
SHBG: Sex hormone-binding globulin
IGF-1: Insulin-like growth factor 1
IGF-1R: Insulin-like growth factor 1 receptors
TCs: Theca cells
CYP17A1: Cytochrome P450c17α
AR: Androgen receptors
CYP19A1: Aromatase
HPO axis: Hypothalamic-pituitary-ovary axis
E: Estrogens
GnRH: Ronadotropin-releasing hormone
LH: Luteinizing hormone
FSH: Follicle-stimulating hormone
ERS: Endoplasmic reticulum stress
GCs: Granulosa cells
TCM: Traditional Chinese Medicine
CMA: Chaperone-mediated autophagy
UPR^^^mt: Unfolded protein response
HMGB1: High mobility group box 1
BOP1: BORC complex subunit BOP1
PAI-1: Plasminogen activator inhibitor-1
CLGI: Chronic low-grade inflammation
CRP: C-reactive protein
IL-18: Interleukin 18
TNF-α: Tumor necrosis factor-alpha
NLR: Neutrophil-to-lymphocyte ratio
hs-CRP: high-sensitivity C-reactive protein
MPV: Mean platelet volume
AMPK: AMP-activated protein kinase
AGEs: Advanced glycation end-products
TG: Triglyceride
LDL-C: Low-density lipoprotein cholesterol
HDL-C: High-density lipoprotein cholesterol
SII: Systemic immune-inflammation index
GRP78: Glucose-regulated protein 78
ATF4: Activating transcription factor 4
IRE1α: Inositol-requiring enzyme 1α
PERK: Protein kinase RNA-like endoplasmic reticulum kinase
SCFAs: Short-chain fatty acids
LPS: Lipopolysaccharide
AKT: Activity of protein kinase B
JNK: c-Jun N-terminal kinase
TLR-4: Toll-like receptor 4
TG: Triglycerides
IFN-γ: Interferon-gamma
IL-22: Interleukin-22
AgRP: Agouti-related protein
GABA: Gamma-aminobutyric acid
GDCA: Glycodeoxycholic acid
TUDCA: Tauroursodeoxycholic acid
NPY: Neuropeptide Y
LEPR: Leptin receptors
GFW: Guizhi Fuling Wan
EA: Electroacupuncture
QGW: Qi Gong Wan
ADM: Adrenomedullin
FBG: Fasting blood glucose
TC: Total cholesterol
GLP-1: Glucagon-Like Peptide-1
TFEL: Total flavonoids of Eucommia ulmoides leaves
